# Primary progressive aphasia: classification of variants in 100 consecutive Brazilian cases

**DOI:** 10.1590/S1980-57642013DN70100017

**Published:** 2013

**Authors:** Mirna Lie Hosogi Senaha, Paulo Caramelli, Sonia M.D. Brucki, Jerusa Smid, Leonel T. Takada, Claudia S. Porto, Karolina G. César, Maria Niures P. Matioli, Roger T. Soares, Letícia L. Mansur, Ricardo Nitrini

**Affiliations:** 1 PhD, Member of Behavioral and Cognitive Neurology Unit of Department of Neurology, University of São Paulo School of Medicine, São Paulo SP, Brazil.; 2 MD, PhD, Professor, Department of Internal Medicine, Faculty of Medicine, Federal University of Minas Gerais, Belo Horizonte, Minas Gerais MG, Brazil.; 3 MD, PhD, Member of Behavioral and Cognitive Neurology Unit of Department of Neurology, University of São Paulo School of Medicine, São Paulo SP, Brazil.; 4 MD, Member of Behavioral and Cognitive Neurology Unit of Department of Neurology, University of São Paulo School of Medicine, São Paulo SP, Brazil.; 5 MD, PhD, Professor, Department of Neurology, University of São Paulo School of Medicine, São Paulo SP, Brazil.

**Keywords:** primary progressive aphasia, clinical consensus, variants, agrammatic, logopenic, semantic, semantic dementia

## Abstract

Primary progressive aphasia (PPA) is a neurodegenerative clinical syndrome characterized primarily by progressive language impairment. Recently, consensus diagnostic criteria were published for the diagnosis and classification of variants of PPA. The currently recognized variants are nonfluent/agrammatic (PPA-G), logopenic (PPA-L) and semantic (PPA-S).

**OBJECTIVE:**

To analyze the demographic data and the clinical classification of 100 PPA cases.

**METHODS:**

Data from 100 PPA patients who were consecutively evaluated between 1999 and 2012 were analyzed. The patients underwent neurological, cognitive and language evaluation. The cases were classified according to the proposed variants, using predominantly the guidelines proposed in the consensus diagnostic criteria from 2011.

**RESULTS:**

The sample consisted of 57 women and 43 men, aged at onset 67.2±8.1 years (range of between 53 and 83 years). Thirty-five patients presented PPA-S, 29 PPA-G and 16 PPA-L. It was not possible to classify 20% of the cases into any one of the proposed variants.

**CONCLUSION:**

It was possible to classify 80% of the sample into one of the three PPA variants proposed. Perhaps the consensus classification requires some adjustments to accommodate cases that do not fit into any of the variants and to avoid overlap where cases fit more than one variant. Nonetheless, the established current guidelines are a useful tool to address the classification and diagnosis of PPA and are also of great value in standardizing terminologies to improve consistency across studies from different research centers.

## Abstract (pt)

A afasia progressiva primária (APP) é uma síndrome clínica neurodegenerativa caracterizada pelo comprometimento predominante e progressivo da linguagem. Recentemente, foi publicado um consenso clínico para o diagnóstico e classificação das variantes da APP. As variantes reconhecidas atualmente são: não-fluente/agramática (APP-G), logopênica (APP-L) e semântica (APP-S).

**OBJETIVO:**

Analisar os dados demográficos e classificar as variantes de uma amostra de 100 casos de APP.

**MÉTODOS:**

Foram analisados os achados de 100 pacientes de APP que foram encaminhados consecutivamente para avaliação fonoaudiológica entre 1999 e 2012. Os pacientes foram submetidos à avaliação neurológica, cognitiva e de linguagem. A partir, principalmente, dos critérios elaborados pelo consenso clinico de APP, os casos foram classificados em uma das variantes.

**RESULTADOS:**

Cem casos, 43 homens e 57 mulheres, foram avaliados. A idade de início variou entre 53 e 83 anos (x=67.2 (±8.1). Foram identificados 35 casos de APP-S, 29 de APP-G e 16 de APP-L. Vinte casos não se enquadraram em nenhumas das três variantes.

**CONCLUSÃO:**

Foi possível classificar distúrbio de linguagem em 80% da amostra em uma das três variantes de APP. A recomendação atual estabelecida pelo consenso clínico é uma ferramenta útil para direcionar a classificação e diagnóstico da APP e também é de grande valor para uniformidade das terminologias entre os diferentes centros de pesquisa. Porém, alguns ajustes seriam interessantes para contemplar os casos que não se encaixam em nenhuma das variantes e para evitar a sobreposição de casos que poderiam se encaixar em mais de uma variante.

## INTRODUCTION

Primary progressive aphasia (PPA) is a neurodegenerative clinical syndrome characterized primarily by progressive language impairment. Systematic studies on PPA started after Mesulam^1^ published his seminal paper entitled "Slowly progressive aphasia without generalized dementia" in 1982. Indeed, cases reported over the past 100 years by Pick, Déjerine, Sérieux and Rosenfeld, which presented degenerative diseases with language disturbances in the initial phase, would possibly fit current criteria for the condition now recognized as PPA.^2^

Intensive research on PPA has been carried out toward gaining a better understanding of several aspects of this syndrome, such as neuroimaging, genetics, neuropathology, clinical and cognitive features.

Regarding the clinical characteristics of PPA, numerous investigations have explored the heterogeneity of the linguistic forms of this syndrome. For some years, there have been different visions and controversies about the classification and diagnosis of this syndrome.^3-6^ However, more recently, consensus diagnostic criteria were published with the objective of providing a standard approach to the diagnosis of PPA and its classification across multicenter studies, by a group of experienced clinicians and researchers from different centers.^7^

The consensus establishes that the classification of PPA variants must be based primarily on the clinical features. Besides the clinical diagnosis (first level), the consensus criteria also establish other two levels for the diagnosis: imaging-supported and definite pathologic diagnosis.

The first level is clinical classification, done in two steps. In the first step, it is determined whether the patient has PPA according to inclusion and exclusion criteria based on Mesulam's guidelines^8-9^ (Table 1). The second step involves the classification of language disturbance into one of the currently recognized variants: nonfluent/agrammatic (PPA-G), logopenic (PPA-L) and semantic (PPA-S), based on language and speech characteristics. For each variant, there are core and ancillary features for the diagnosis (Table 2).

**Table 1:** Inclusion and exclusion criteria for PPA diagnosis: based on criteria by Mesulam^8,9^ (Gorno-Tempini et al., 2011).^7^

**Inclusion: all above criteria must be answered positively**	
1.	Most prominent clinical feature is a language disturbance;
2.	The language disturbance is the principal cause of impaired daily living activities;
3.	Aphasia should be the most prominent deficit at symptom onset, for the initial phases of the disease.
**Exclusion: all above criteria must be answered negatively**	
1.	Pattern of deficits is better accounted for by other nondegenerative nervous system or medical disorders;
2.	Cognitive disturbance is a better accounted for by psychiatric diagnosis;
3.	Prominent initial episodic memory loss, visual memory and visuospatial impairments;
4.	Prominent, initial behavioral disturbance.

**Table 2:** Guidelines to classify PPA variants according to recommendations in consensus diagnosis criteria (Gorno-Tempini et al., 2011).^7^

**PPA-G - Nonfluent/agrammatic variant (also known as progressive nonfluent aphasia)**	
A.	One osf the following core features must be present:
1.	Agrammatism in language production;
2.	Effortful, halting speech with inconsistent speech sound errors and distortions (apraxia of speech).
B.	At least 2 of 3 of the following ancillary features must be present:
1.	Impaired comprehension of syntactically complex (non-canonical) sentences;
2.	Spared single-word comprehension;
3.	Spared object knowledge.
**PPA-S - Semantic variant (also known as semantic dementia)**	
A.	Both of the following core features must be present:
1.	Impaired object naming;
2.	Impaired single-word comprehension.
B.	At least 3 of 4 of the following ancillary features must be present:
1.	Impaired object knowledge, particularly for low-frequency or low-familiarity items;
2.	Surface dyslexia or dysgraphia;
3.	Spared repetition;
4.	Spared grammaticality and motor aspects of speech.
**PPA-L - Logopenic variant (also known as logopenic progressive aphasia)**	
A.	Both of the following core features must be present:
1.	Impaired single-word retrieval in spontaneous speech and naming;
2.	Impaired repetition of phrases and sentences.
B.	At least 3 of 4 of the following ancillary features must be present:
1.	Phonological errors (phonemic paraphasias) in spontaneous speech or naming;
2.	Spared single-word comprehension and object knowledge;
3.	Spared motor speech;
4.	Absence of frank agrammatism.

The second level of classification, besides the clinical findings of the characteristics of each subtype, includes imaging-supported diagnosis, where the following features must be present on structural or functional neuroimaging: [1] nonfluent/agrammatic variant (PPA-G), predominant left fronto-insular area abnormalities on neuroimaging assessment; [2] semantic variant, predominant anterior temporal lobe atrophy, hypoperfusion or hypometabolism; and [3] logopenic variant, imaging abnormalities predominantly in left posterior perisylvian or parietal areas are necessary.

For the third classification level, definite pathologic diagnosis, the consensus establishes that, besides the typical clinical characteristics of each variant, the patient must present pathologic or genetic mutations definitely associated with the frontotemporal lobar degeneration (FTLD) spectrum, Alzheimer's disease (AD), or another specific etiology.

Studies have shown that PPA-G is more frequently associated with deposits of hyperphosphorylated tau protein, whereas PPA-S is associated with deposits of ubiquitinated TDP-43 protein, whereas PPA-L is more frequently caused by AD, with beta-amyloid and tau protein parenchymal aggregation and deposition.^10-15^

Due to the lack of biological markers, PPA clinical classification into one of the variants may help the clinician in identifying the possible pathologic basis and may also assist in the choice of pharmacological intervention.

The aim of the present study was to analyze the demographic data and the clinical classification of 100 consecutive PPA patients evaluated in Brazil.

## METHODS

Demographic data from a series of 100 PPA patients consecutively evaluated between 1999 and 2012 were analyzed.

All patients underwent neurological examination, neuroimaging assessment and comprehensive cognitive, language and semantic memory evaluation. All patients were submitted to structural (MRI/CT) neuroimaging assessment and/or functional (SPECT/PET scan) neuroimaging assessment.

All patients were also submitted to language evaluation by the same speech pathologist (MLHS), which included application of the following tools: communication functional evaluation, aphasia battery tests (Beta MT-86,^16^ Boston Diagnostic Aphasia Exam-BDAE,^17^ Boston Naming Test,^18^ Human Frontier Science Program (HFSP) reading and writing protocols^19^ and in some cases additional evaluation (example: tasks of semantic memory battery previously described in other papers).^20-21^

To evaluate the core and ancillary criteria for diagnosis of the PPA variants, a number of different tasks were used. The analyses of oral production in spontaneous speech and on the Boston Cookie theft picture description task^17^ were employed for syntax assessment. In some cases, there was an additional task of ordering single written words, printed on a separated card, to constitute a correct sentence. Syntactic comprehension was evaluated through matching tasks (sentences-pictures) from the Beta MT-86 protocol.^16^ Speech motor disturbances were evaluated from the assessment proposed by Darley et al.^22^ Semantic comprehension was evaluated through word-picture matching and word definition tasks from the semantic memory battery.^20-21^ The BDAE^17^ stimulus were used to assess sentence repetition. Object knowledge (visual semantic memory) was assessed through qualitative analysis of responses on the Boston Naming Test^18^ and in some cases the Peno protocol,^23^ Pyramids and Palm Tree (PPT)^24^ and famous face recognition tests were used.^20,21^ Written language abilities were examined through reading aloud and dictation tasks from the HFSP protocol^19^ or Beta MT-86 aphasia protocol".^16^

From the data obtained in the language evaluation, cases were classified according to the proposed variants, using predominantly the criteria proposed by the consensus^7^ (Table 1), but also with support of other studies.^25-28^ Besides the 100 consecutive PPA cases included in this study over the 13-year study period, two patients evaluated were diagnosed with progressive apraxia of speech based on current criteria.

## RESULTS

The evaluated sample comprised 57 women and 43 men. The main demographic data and Mini-Mental State Examination (MMSE) scores^29,30^ are shown in Table 3. Ninety-eight patients were right-handed, one case had a history of developmental dyslexia and two cases had a history of transient global amnesia episodes before the emergence of language disturbance.

**Table 3:** Demographic data of PPA patients studied.

		Mean (SD)	Median	Minimum/maximum values
**All PPA patients (n=100; 57 women and 43 men)**	Age	69.8 (8.3)	69.5	54-90
	Age at onset	67.2 (8.1)	67.0	53-83
	Educational level	12.5 (4.7)	15	2-20*
	Mini-Mental State Exam	22.1 (4.8)	23	11-29**
**PPA-S (n=35; 18 women and 17 men)**	Age	69.1 (8.0)	68.0	55-88
	Age at onset	64.1 (13.6)	67.0	53-82
	Educational level	13.5 (4.5)	15	3-20
	Mini-Mental State Exam	21.6 (5.5)	23	11-29***
**PPA-G (n=29; 18 women and 11 men)**	Age	70.1 (8.6)	70.0	57-85
	Age at onset	68.2 (8.5)	68.0	54-83
	Educational level	11.4 (4.9)	11	2-20
	Mini-Mental State Exam	22.2 (5.0)	23.5	11-20****
**PPA-L (n=16; 10 women and 6 men)**	Age	67.4 (8.3)	68.5	54-79
	Age at onset	65.3 (8.5)	66.5	51-77
	Educational level	13.7 (3.6)	15	5-20
	Mini-Mental State Exam	20.6 (4.5)	24	13-29*****

SD, standard deviation;

* one case of informal education;

** data from 88 cases;

*** 29 cases;

**** 26 cases;

***** 15 cases.

**Figure 1:**
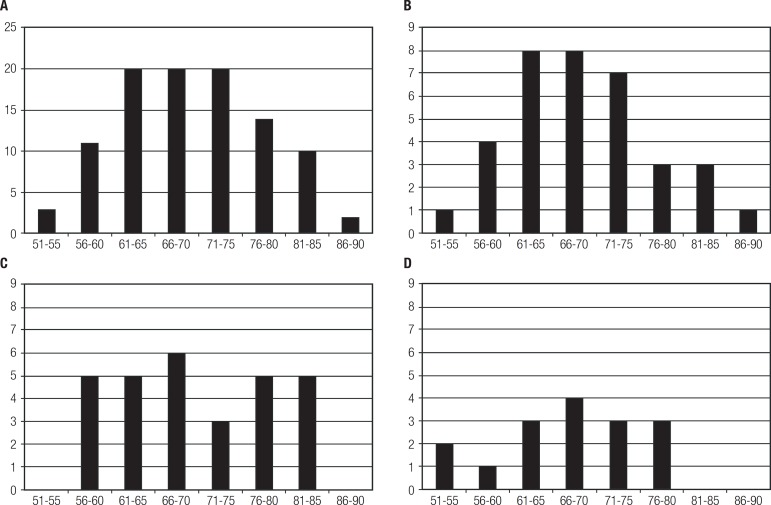
[A] Distribution of age at diagnosis for all PPA cases. [B] Distribution of age at diagnosis for PPA-S cases. [C] Distribution of age at diagnosis for PPA-G cases. [D] Distribution of age at diagnosis for PPA-L cases.

Figure 1 shows age distribution at time of diagnosis for the overall sample and for the three main variants. Age at diagnosis ranged from 54 to 90 years. Thirty-four patients had disease onset before 65 years of age.

Classification into the PPA variants of the 100 cases can be seen in Figure 2. It was possible to classify 80% of the sample into one of the three PPA variants proposed by the International consensus:^7^ 35 presented PPA-S, 29 PPA-G, and 16 PPA-L. Thus, it was not possible to classify 20% of the cases into any one of the proposed variants.

**Figure:**
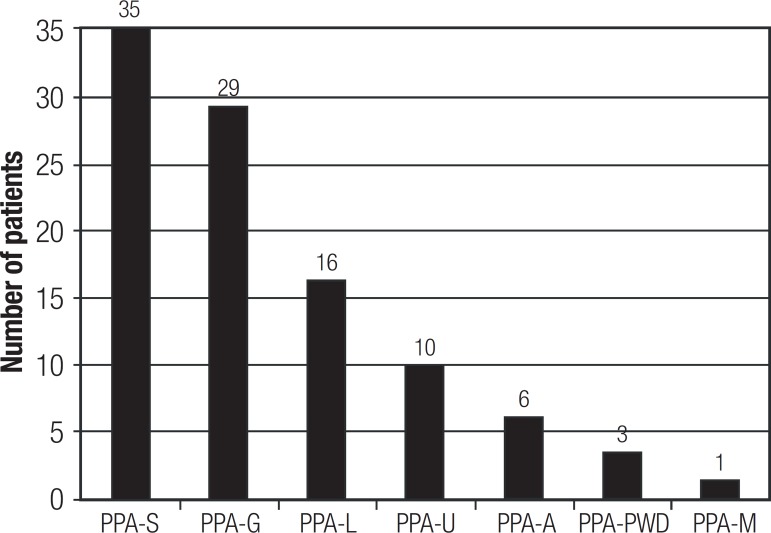
PPA: primary progressive aphasia; PPA-S: semantic variant; PPA-G: nonfluent/agrammatic variant; PPA-L: logopenic variant; PPA-U: unclassifiable; PPA-A: anomic variant; PPA-PWD: pure word deafness variant; PPA-M: mixed variant.^28,29^

Among these 20 unclassifiable cases, there were six cases of anomic aphasia (PPA-A), three of pure word deafness (PPA-PWD), one case of mixed PPA (PPA-M)^31,32^ and ten unclassifiable cases (PPA-U).

Table 4 shows the performance on language tasks. Table 5 shows age at onset and at evaluation along with structural and/or functional neuroimaging findings for all PPA cases.

**Table 4:** Performance on language tasks of PPA patients.

Case	Agrammatism in spontaneous speech	Motor speech disorders	Semantic comprehension		Syntax comprehension (Montréal-Tolouse MT-86^16^)	Picture naming (BNT^18^)(60 items)	Fluency		Sentence repetition (BDAE^17^)(16 sentences)	Surface dyslexia and/or surface dysgraphia
			**Word-picture matching**	**Word definition (13 or 33 items)**	**Sentence-picture matching (38 items)**		**Category (animals)**	**Letter (F, A, S)**		
PPA-S 1	no	no	65.5% (SMB)	53.8% (13)	52.6%	5 (8.3%)	0	3	6.8%	yes
PPA-S 2	no	no	95.5% (SMB)	78.8% (33)	100.0%	23 (38.3%)	10	22	93.8%	yes
PPA-S 3	no	no	88.9% (SMB)	42.4% (33)	97.4%	13 (21.7%)	5	17	81.3%	yes
PPA-S 4	no	no	52.2% (SMB)	30.8% (13)	97.4%	3 (5.0%)	2	8	68.8%	yes
PPA-S 5	no	no	62.2% (SMB)	15.4% (13)	97.4%	10 (16.7%)	5	20	62.5%	yes
PPA-S 6	no	no	82.2% (SMB)	7.7% (13)	100.0%	10 (16.7%)	4	25	81.2%	yes
PPA-S 7	no	no	95.5% (SMB)	72.7% (33)	100%	27 (45.0%)	9	33	62.5%	yes
PPA-S 8	no	no	96.7% (SMB)	57.6% (33)	94.7%	30 (50.0%)	7	15	75.0%	yes
PPA-S 9	no	no	82.2% (SMB)	NA	86.8%	3 (5.0%)	3	7	16.3%	possibly(low education)
PPA-S 10	no	no	64.4% (SMB)	30.5% (13)	100.0%	16 (26.7%)	6	13	93.8%	yes
PPA-S 11	no	no	92.2% (SMB)	72.7% (33)	100.0%	41 (68.3%)	19	24	93.8%	yes
PPA-S 12	no	no	85.5% (SMB)	69.2% (13)	100.0%	9 (15.0%)	4	4	68.8%	yes
PPA-S 13	no	no	55.5% (SMB)	30.7% (13)	86.8%	5 (8.3%)	0	12	50.0%	foreigner
PPA-S 14	no	no	75.6% (SMB)	18.2% (33)	100%	7 (11.7%)	6	17	68.8%	yes
PPA-S 15	no	no	73.3% (SMB)	NA	94.7%	5 (8.3%)	2	4	50.0%	yes
PPA-S 16	no	no	40.0% (SMB)	15.1% (33)	97.4%	4 (6.7%)	0	12	87.5%	yes
PPA-S 17	no	no	84.4% (SMB)	61.5% (13)	92.1%	21 (35.0%)	8	17	81.2%	possibly(low education)
PPA-S 18	no	no	71.1% (SMB)	NA	97.4%	5 (8.3%)	6	13	75.0%	no (intense dyslexia and dysgraphia)
PPA-S 19	no	no	63.3% (SMB)	NA	76.3%	10 (16.7%)	1	3	43.8%	yes
PPA-S 20	no	no	63.3% (SMB)	NA	70.2%	1 (1.7%)	1	3	62.5%	yes
PPA-S 21	no	no	85.5% (SMB)	NA	100.0%	9 (15.0%)	4	22	87.5%	yes
PPA-S 22	no	no	42.2% (SMB)	3.0% (33)	94.7%	5 (8.3%)	2	9	75.0%	yes
PPA-S 23	no	no	62.5% (SMB)	NA	81.6%	4 (6.7%)	1	13	50.0%	yes
PPA-S 24	no	no	10.0% (SMB)	NA	34.2%	3 (5.0%)	0	0	37.5%	yes
PPA-S 25	no	no	87.5% (SMB)	46.2% (13)	66.7%	9 (15.0%)	4	13	75.0%	yes
PPA-S 26	no	no	98.9% (SMB)	78.8% (33)	100.0%	20 (33.3%)	10	22	87.5%	yes
PPA-S 27	no	no	76.7% (SMB)	57.60%	94.7%	17 (28.3%)	1	12	93.8%	yes
PPA-S 28	no	no	93.3% (SMB)	60.6% (33)	100.0%	13 (21.7%)	4	33	100.0%	yes
PPA-S 29	no	no	62.2% (SMB)	46.1% (13)	94.7%	5 (8.3%)	4	4	87.5%	yes
PPA-S 30	no	no	92.2% (SMB)	30.7% (13)	100.0%	15 (25.0%)	8	25	87.5%	yes
PPA-S 31	no	no	65.5% (SMB)	NA	92.1%	6 (10.0%)	2	21	43.8%	yes
PPA-S 32	no	no	87.7% (SMB)	78.8% (33)	92.1%	32 (53.3%)	5	6	93.8%	yes
PPA-S 33	no	no	97.8% (SMB)	63.6% (33)	84.2%	27 (45.0%)	12	14	87.5%	yes
PPA-S 34	no	no	67.7% (SMB)	48.5% (33)	92.1%	16 (26.7%)	9	31	100.0%	yes
PPA-S 35	no	no	80.0% (SMB)	69.7% (33)	97.4%	32 (53.3%)	8	18	75.0%	yes
PPA-L 1	no	no	96.7% (SMB)	97.0% (33)	86.8%	43 (71.7%)	7	17	75.0%	yes
PPA-L 2	no	no	95.4% (MT-86)	97.0% (33)	86.8%	43 (71.7%)	7	19	37.5%	yes
PPA-L 3	no	no	95.5% (SMB)	NA	100.0%	70/90 (77.8%)*	9	29	62.5%	yes
PPA-L 4	no	no	100.0% (SMB)	93.9% (33)	71.1%	38 (63.3%)	9	7	81.3%	no
PPA-L 5	no	no	96.7% (SMB)	NA	76.3%	16 (26.7%)	6	3	18.8%	no
PPA-L 6	no	no	95.4% (MT-86)	NA	100.0%	54 (90.0%)	16	30	68.7%	yes
PPA-L 7	no	no	95.5% (SMB)	92.3% (13)	92.1%	32 (58.3%)	13	45	75.0%	yes
PPA-L 8	no	no	100.0% (SMB)	97.0% (33)	100.0%	42 (70.0%)	11	26	87.5%	yes
PPA-L 9	no	no	93.3% (SMB)	92.3% (13)	57.9%	33 (55.0%)	14	2	66.7%	yes
PPA-L 10	no	no	100.0% (SMB)	97.0% (33)	94.7%	57 (95.0%)	15	25	62.5%	yes
PPA-L 11	no	no	100.0% (MT-86a)	NA	78.9%	11 (18.3%)	6	5	25.0%	no
PPA-L 12	no	no	96.7% (SMB)	97.0% (33)	76.3%	53 (88.3%)	6	7	81.3%	no
PPA-L 13	no	no	90.0% (SMB)	NA	76.3%	17 (28.3%)	7	11	56.3%	no
PPA-L 14	no	no	95.0% (SMB)	93.9% (33)	78.9%	44 (73.3%)	5	5	50.0%	no
PPA-L 15	no	no	100.0% (MT-86)	100.0% (13)	52.6%	15 (25.0%)	3	10	81.3%	yes
PPA-L 16	no	no	100.0% (SMB)	90.9% (33)	65.7%	46 (76.7%)	11	9	25.0%	yes
PPA-G 1	no	yes	92.2% (SMB)	NA	71.0%	21 (35.0%)	3	6	12.5%	no
PPA-G 2	yes	no	93.3% (SMB)	NA	86.8%	25 (41.7%)	4	3	62.5%	no
PPA-G 3	yes	yes	81.1% (SMB)	NA	52.6%	25 (41.7%)	5	1	56.3%	no
PPA-G 4	no	yes	100.0% (MT-86)	NA	84.2%	37 (61.7%)	6	5	62.5%	no
PPA-G 5	yes	no	93.3% (SMB)	100.0% (13)	73.7%	48 (80.0%)	3	4	62.5%	yes
PPA-G 6	no	yes	90.0% (SMB)	72.7% (33)	57.9%	39 (65.0%)	6	17	87.5%	yes
PPA-G 7	no	yes	95.4% (MT-86)	NA	68.4%	15 (25.0%)	2	4	31.3%	no
PPA-G 8	yes	yes	97.8% (SMB)	93.9% (33)	57.9%	32 (53.3%)	8	3	25.0%	no
PPA-G 9	no	yes	100.0% (MT-86)	NA	71.1%	40 (66.7%)	2	9	56.3%	no
PPA-G 10	yes	yes	100.0% (SMB)	97.0% (33)	92.1%	43 (71.7%)	14	22	68.8%	yes
PPA-G 11	no	yes	95.4% (MT-86)	NA	92.1%	27 (45.0%)	6	5	75.0%	foreigner
PPA-G 12	yes	yes	95.4% (MT-86)	NA	84.2%	43 (71.7%)	7	15	50.0%	yes
PPA-G 13	yes	yes	95.4% (MT-86)	87.9% (33)	89.5%	44 (73.3%)	14	21	37.5%	no
PPA-G 14	yes	yes	100.0% (SMB)	NA	89.5%	30 (50.0%)	7	NA	50.0%	foreigner
PPA-G 15	yes	no	95.0% (SMB)	75.6% (33)	68.4%	25 (41.7%)	18	8	12.5%	no
PPA-G 16	yes	yes	94.1% (SMB)	NA	55.3%	14 (23.3%)	3	4	12.5%	no
PPA-G 17	yes	yes	98.9% (SMB)	81.8% (33)	73.7%	24 (40.0%)	11	10	31.3%	yes
PPA-G 18	yes	yes	97.8% (SMB)	63.3% (13)	50.0%	37 (61.7%)	3	1	68.8%	no
PPA-G 19	yes	yes	100.0% (MT-86)	NA	92.1%	43 (71.7%)	8	8	81.3%	no
PPA-G 20	yes	yes	100.0% (SMB)	NA	81.6%	30 (50.0%)	11	1	75.0%	low education
PPA-G 21	yes	no	97.8% (SMB)	90.9% (33)	76.3%	44 (73.3%)	11	13	100.0%**	yes
PPA-G 22	yes	yes	88.9% (SMB)	NA	57.9%	17 (28.3%)	4	3	6.3%	no
PPA-G 23	yes	yes	100.0% (SMB)	NA	76.3%	35 (58.3%)	3	4	62.5%	yes
PPA-G 24	no	yes	91.1% (SMB)	72.7% (33)	100.0%	32 (53.3%)	9	11	87.5%	no
PPA-G 25	yes	no	95.5% (SMB)	NA	65.8%	26 (43.3%)	2	1	75.0%	no
PPA-G 26	no	yes	94.4% (SMB)	93.9% (33)	89.5%	34 (56.7%)	10	15	52.5%	no
PPA-G 27	yes	no	96.7% (SMB)	NA	100.0%	39 (65.0%)	8	15	100.0%	no
PPA-G 28	no	yes	76.2% (SMB)	51.5% (33)	68.4%	15 (25.0%)	3	1	50.0%	low education
PPA-G 29	yes	yes	100.0% (SMB)	100% (13)	84.2%	51 (85.0%)	13	6	31.3%	no
PPA-A 1	no	no	100.0% (SMB)	84.8% (33)	94.7%	43 (71.7%)	11	33	93.8%	yes
PPA-A 2	no	no	95.4% (MT-86)	100.0% (13)	100.0%	42 (70.0%)	9	17	100.0%	yes
PPA-A 3	no	no	100.0% (MT-86)	NA	100.0%	16/25 (64.0%)**	6	NA	100.0%**	no
PPA-A 4	no	no	96.7% (SMB)	NA	89.5%	48 (80.0%)	14	31	100.0%	no
PPA-A 5	no	no	100.0% (SMB)	100.0% (33)	100.0%	39 (65.0%)	8	29	100.0%	yes
PPA-A 6	no	no	100.0% (MT-86)	NA	89.5%	32 (53.3%)	10	11	93.8%	no
PPA-M 1	yes	yes	73.3% (SMB)	NA	89.5%	13 (21.7%)	8	5	43.8%	yes
PPA-PWD 1	no	no	100.0% (SMB)	NA	100.0%	58 (96.7%)	12	43	37.5%	no
PPA-PWD 2	no	no	100.0% (SMB)	NA	52.6%	49 (81.7%)	10	14	0.0%	yes
PPA-PWD 3	no	yes	100.0% (MT-86)	NA	52.6%	29 (48.3%)	12	17	0.0%	no
PPA-U 1	no	no	93.3% (SMB)	69.2% (13)	84.2%	18 (30.0%)	5	14	31.3%	possibly(low education)
PPA-U 2	no	no	90.9% (MT-86)	NA	84.2%	10 (16.7%)	6	17	50.0%	possibly(low education)
PPA-U 3	no	no	95.5% (SMB)	NA	94.7%	34 (56.7%)	10	31	75.0%	yes
PPA-U 4	dyssynntaxia	no	100.0% (SMB)	NA	86.8%	26 (43.3%)	8	41	43.8%	yes
PPA-U 5	no	no	81.1% (SMB)	72.7% (33)	73.7%	21 (35.0%)	4	10	12.5%	low education
PPA-U 6	jargon	no	73.3% (SMB)	NA	44.7%	0 (0%)	0	NA	12.5%	no
PPA-U 7	no	no	94.4% (SMB)	NA	76.3%	29 (48.3%)	3	9	68.8%	yes
PPA-U 8	no	no	88.9% (SMB)	75.7% (33)	76.3%	33 (55.0%)	6	15	50.0%	yes
PPA-U 9	no	no	80.0% (SMB)	45.5% (33)	63.1%	13 (21.7%)	3	9	37.5%	low education
PPA-U 10	jargon	no	72.2% (SMB)	NA	57.9%	7 (11.7%)	1	3	18.8%	no

BNT: Boston Naming Test; BDAE: Boston Diagnostic Aphasia Examination; SMB: semantic memory battery; MT-86: Montréal-Tolouse MT-86; NA: data not available;

* SMB (semantic memory battery;

** MT-86 (Montréal-Tolouse MT-86).

**Table 5:** Neuroimaging findings, age at onset, and age at evaluation of PPA patients.

Case	Age at onset	Age at evaluation	Structural and/or Functional Neuroimaging findings
PPA-S 1	61	66	frontotemporal atrophy and hypoperfusion and parietal hypoperfusion, predominantly left
PPA-S 2	56	59	left temporal atrophy and hypoperfusion and left perisylvian enlargement
PPA-S 3	62	63	anterior temporal hypoperfusion, predominantly right
PPA-S 4	57	62	left temporal atrophy
PPA-S 5	53	55	temporal atrophy ,predominantly right
PPA-S 6	64	68	temporal atrophy, predominantly left
PPA-S 7	64	67	no focal atrophy; left temporal hypoperfusion
PPA-S 8	64	65	severe left temporal and mild left frontal hypoperfusion
PPA-S 9	80	82	left temporal atrophy
PPA-S 10	76	77	left frontotemporal hypoperfusion
PPA-S 11	66	67	left temporal atrophy and left temporal hypoperfusion with frontal extension
PPA-S 12	82	83	left anterior temporal atrophy and left temporal hypoperfusion
PPA-S 13	78	80	mild global atrophy ; temporal anterior hypoperfusion, predominantly left
PPA-S 14	54	56	anterior temporal atrophy, predominantly left
PPA-S 15	57	59	left anterior temporal atrophy
PPA-S 16	70	73	anterior temporal atrophy and hypometabolism
PPA-S 17	66	71	left temporal atrophy and hypoperfusion
PPA-S 18	72	75	anterior temporal atrophy and hypoperfusion, predominantly left
PPA-S 19	73	88	left anterior temporal atrophy and hypoperfusion, and mild right frontal hypoperfusion
PPA-S 20	53	58	temporal atrophy, predominantly left, and left anterior temporal hypoperfusion with mild frontal extension
PPA-S 21	62	65	temporal atrophy, predominantly right
PPA-S 22	69	72	anterior temporal and perisylvian atrophy, predominantly left, and left temporal hypoperfusion
PPA-S 23	68	72	temporal atrophy, predominantly left, and left frontotemporoparietal hypoperfusion
PPA-S 24	62	67	temporal atrophy, predominantly left
PPA-S 25	79	81	temporal atrophy, predominantly left, and left temporoparietal hypoperfusion
PPA-S 26	64	69	anterior temporal atrophy, predominantly left
PPA-S 27	68	70	temporal hypoperfusion, predominantly left
PPA-S 28	60	63	left anterior temporal atrophy and left temporal with frontal extension hypoperfusion
PPA-S 29	67	69	left temporal atrophy and hypoperfusion
PPA-S 30	62	64	left temporal atrophy and temporoparietal hypoperfusion
PPA-S 31	63	65	temporal atrophy and hypoperfusion, predominantly left
PPA-S 32	75	78	left frontoparietal hypometabolism
PPA-S 33	61	63	left anterior temporal atrophy and hypoperfusion
PPA-S 34	69	72	anterior temporal and frontal atrophy, predominantly left
PPA-S 35	72	74	bilateral anterior temporal and amygdala atrophy, and frontotemporal and cingulate hypoperfusion (R>L)
PPA-L 1	57	61	left frontotemporoparietal hypoperfusion and right frontal hypoperfusion
PPA-L 2	77	79	no atrophy, superior posterior parietal hypoperfusion
PPA-L 3	72	74	lateral ventricle enlargement, left more than right.
PPA-L 4	67	68	left superior frontal hypoperfusion
PPA-L 5	76	79	left posterior frontal and temporoparietal hypoperfusion
PPA-L 6	65	67	left temporal atrophy and hypoperfusion
PPA-L 7	77	78	mild global atrophy and left temporal hypometabolism
PPA-L 8	69	71	left temporal atrophy and left frontotemporal hypoperfusion
PPA-L 9	55	57	no atrophy and left frontal hypoperfusion
PPA-L 10	61	62	left temporoparietal hypometabolism
PPA-L 11	72	74	predominantly left perisylvian atrophy
PPA-L 12	67	69	left temporal atrophy and left frontotemporoparietal and right frontal hypometabolism
PPA-L 13	53	55	left temporal and hippocampal atrophy and left frontotemporoparietal hypoperfusion
PPA-L 14	66	69	diffuse mild atrophy slightly more evident at left temporal lobe
PPA-L 15	60	62	mild left perisylvian atrophy and predominantly left temporoparietal hypoperfusion
PPA-L 16	51	54	left hypoperfusion
PPA-G 1	73	76	left temporal atrophy and left temporoparietal hypoperfusion
PPA-G 2	74	78	left superior parietal hypoperfusion
PPA-G 3	65	68	bilateral frontotemporal (inferior and anterior) hypoperfusion
PPA-G 4	82	82	lateral ventricle asymmetry, (L>R).
PPA-G 5	64	65	right frontal and left parietal hypoperfusion
PPA-G 6	70	74	left temporal atrophy and left frontotemporal hypoperfusion
PPA-G 7	70	73	right Sylvian fissure enlargement, right frontotemporoparietal hypoperfusion
PPA-G 8	61	62	left perisylvian atrophy
PPA-G 9	60	63	left frontotemporoparietal hypoperfusion
PPA-G 10	81	82	predominantly posterior cortical atrophy
PPA-G 11	71	72	mild global atrophy and left temporoparietooccipital hypoperfusion
PPA-G 12	68	70	mild cortical atrophy and predominantly right frontal hypoperfusion
PPA-G 13	56	57	left temporoparietal atrophy and bilateral parietooccipital hypoperfusion
PPA-G 14	71	77	mild cortical atrophy and left temporal hypoperfusion
PPA-G 15	83	85	left temporoparietal hypometabolism
PPA-G 16	82	83	frontotemporal and hippocampal atrophy, predominantly left, and predominantly left frontal and cingulated hypoperfusion
PPA-G 17	58	60	left temporoparietal hypoperfusion
PPA-G 18	68	70	cortical atrophy, predominantly left frontotemporal hypoperfusion
PPA-G 19	58	59	sulcal and ventricular enlargement (L>R) and left frontal and left temporal (perisylvian) hypoperfusion
PPA-G 20	74	76	no focal atrophy (CT)
PPA-G 21	67	67	left perisylvian atrophy and predominantly left frontotemporal hypoperfusion
PPA-G 22	60	62	fronto-temporal atrophy, predominantly left, and left parietal and mesial temporal hypoperfusion
PPA-G 23	67	69	mild left perisylvian atrophy and left anterior cingulated hypoperfusion
PPA-G 24	74	77	lateral ventricle enlargement, predominantly left, and left temporoparietal hypoperfusion
PPA-G 25	54	57	global atrophy, predominantly left
PPA-G 26	59	61	lateral ventricle enlargement (L>R) and temporoparietal hypoperfusion, predominantly left
PPA-G 27	58	59	perisylvian atrophy (right more than left) and right frontotemporal hypoperfusion
PPA-G 28	81	82	frontotemporal hypoperfusion, predominantly left
PPA-G 29	67	69	frontotemporal atrophy; frontoparietotemporal hypoperfusion, predominantly right
PPA-A 1	69	70	left temporal hypoperfusion
PPA-A 2	64	64	left temporal (perisylvian) atrophy and left temporoparietal (posterior) hypoperfusion
PPA-A 3	73	75	left temporal atrophy
PPA-A 4	71	72	MRI: no asymmetries, and left medial temporal hypoperfusion
PPA-A 5	73	76	temporoparietal hypoperfusion
PPA-A 6	83	84	bilateral temporal hypoperfusion
PPA-M 1	69	79	global mild atrophy and predominantly left temporoparietal hypoperfusion
PPA-PWD 1	59	63	inspecific signals, predominantly left
PPA-PWD 2	61	63	no atrophy; right temporoparietal and very mild left superior posterior parietal hypoperfusion
PPA-PWD 3	67	71	predominantly frontotemporal atrophy
PPA-U 1	71	72	no atrophy; left temporal hypoperfusion
PPA-U 2	68	71	mild global atrophy; left more than right hippocampal atrophy, and left temporoparietal hypoperfusion
PPA-U 3	69	75	left perisylvian atrophy and left frontotemporoparietal hypoperfusion
PPA-U 4	61	65	left temporoparietal atrophy
PPA-U 5	71	75	left perisylvian atrophy and bilateral anterior temporal atrophy
PPA-U 6	80	90	global atrophy; left frontal hypoperfusion
PPA-U 7	67	67	frontotemporal atrophy and hypoperfusion, predominantly left
PPA-U 8	55	56	sulcal enlargement on left side, and left parietooccipital hypoperfusion
PPA-U 9	83	84	global atrophy and left temporoparietal hypoperfusion
PPA-U 10	77	79	left cortical atrophy and left temporal hypoperfusion with frontoparietal extension

Observation: Cases with less than 2-year history of progressive language disturbances were followed and PPA diagnosis confirmed.

## DISCUSSION

The data reported in this study included a large number of consecutive PPA cases submitted to language evaluation by the same speech pathologist over a period of 13 years. Our sample had higher mean age at onset than that reported in previous studies^10,33-36^ and also contained more women.^33,34^

Rogalsky et al.^37^ suggested that learning disabilities (LD) may constitute a risk factor for PPA. Their results showed that patients with PPA and their first degree relatives had a higher frequency of LD, especially dyslexia, when compared to patients with AD, with behavioral variant of FTLD and to healthy older adults.

During history taking, we systematically asked the patient and informant whether the patient had a history of LD, or difficulty with reading or writing during the first years of school. The informant was in most cases the patient's spouse or son/daughter, who clearly had not lived with the patient during this period. We did not investigate whether any of the patients' first-degree relatives had LD. A history of developmental dyslexia was found in only one case.

Recently, a possible association between transient global amnesia and PPA was reported.^38,39^ The two patients cited in the Nitrini et al.^39^ study are included in this sample.

Different manifestations of FTLD in its behavioral and language variants frequently occur in the pre-senile phase. FTLD is considered the second most common cause of degenerative dementia after AD in subjects between 45 and 65 years of age, being less prevalent in the elderly.^40-45^ However, this association of FTLD and pre-senile onset must be viewed with caution, because some investigators have reported a significant number of FTLD cases with an age of onset beyond 65.^13,46^ Indeed, in a demographic study of 100 semantic dementia cases, Hodges et al. verified that the age at diagnosis was over 65 years in 46% of the patients.^13^

Considering all three PPA variants defined by the clinical consensus, semantic and nonfluent/agrammatic cases prevailed in our sample with logopenic cases proving less frequent. Studies conducted after the publication of the clinical consensus have presented different proportions of these variants. A very small number of PPA-L cases in relation to other variants were found in the study by Sajjadi et al. with only 4.3% of patients being classified as PPA-L versus 28.3% as PPA-S and 26.1% as PPA-G.^46^ In another report by Thompson et al. investigating fluency and agrammatism in PPA, 37 consecutive cases from the Northwestern Cognitive Neurology and Alzheimer Disease Center were recruited between 2007 and 2010 and a smaller number of PPA-S (6) than PPA-G (11) or PPA-L (20) cases was found.^36^ Leyton et al. recruited 47 consecutive PPA patients and found 14 PPA-S, 15 PPA-G and 18 PPA-L^15^ cases. In another study, conducted prior to the consensus publication and considering only the dichotomy semantic dementia (SD) versus nonfluent progressive aphasia (NFPA), Hodges et al. found a larger number of SD. In the cited study, the authors included all new patients who were examined at the Memory Clinic in Cambridge, UK, between 1990 and 2007.13 Three thousand six-hundred new patients were evaluated and 416 received the diagnosis of focal cortical dementia. Of these 416 cases, 128 received the diagnosis of fronto-temporal dementia (FTD), 110 SD, 66 NFPA, with 36 mixed (mixed aphasic/behavioural cases) and 66 corticobasal syndrome cases.

Further studies on the clinical classification of the PPA subtypes using the guidelines of the consensus may determine the real frequency of each variant.

Among the three PPA variants, we considered PPA-S and PPA-G to have more consistent profiles in relation to language characteristics. In PPA-S, the word comprehension and anomia due to semantic degradation are the core and necessary symptoms for the diagnosis of this variant. These symptoms are noticeable aspects of the PPA-S profile. In cases of very mild PPA-S, these symptoms may not be so evident, however when more sensitive tasks are used with less familiar stimuli, the diagnosis of this variant is possible. The word-definition task, requiring besides the stimulus categorization, detailed information that defines the lexical item more precisely, was shown to be a more sensitive task than the word-picture matching task (with eight alternatives of the same semantic category) for detecting verbal semantic memory impairment.^21^

In addition to the two core symptoms mentioned for the classification and diagnosis of PPA-S, the presence of surface dyslexia and/or surface dysgraphia, ancillary symptoms for the diagnosis of PPA-S, is also very frequent, since this is secondary to the verbal semantic memory impairment. Besides surface dyslexia, some cases who were able to read by lexical processing (without regularization errors), but who could not access the meaning, have been previously described.^47,48^ In other words, in this form of dyslexia, called semantic dyslexia, patients can read irregular words correctly but without comprehension.^47^ Other symptoms of PPA-S which are not cited in the classification scheme, but may help in the diagnosis of this variant, are: dissociation between semantic and syntactic comprehension, oral production fluency in quantitative terms, greater difficulty on semantic fluency tasks than phonological fluency tasks (for example, FAS) and low score in confrontation naming. The sum of these symptoms, and the interpretation of these linguistic data, resulting from verbal semantic memory impairment facilitate the diagnosis of PPA-S. In our experience, there is a variation related to the concomitance of verbal and nonverbal semantic impairment. The intensity of the nonverbal semantic impairment varied among our cases, independently of verbal semantic impairment intensity.^20,21^

Regarding PPA-G, the two core characteristics according to the clinical consensus - agrammatism in language production and speech motor problems - also constitute exclusive symptoms of this variant that allow its identification. However, according to the clinical consensus, these two central characteristics can occur together or separately, allowing different clinical manifestations to be included under the terminology of PPA-G. Therefore, PPA-G can be considered: [1] patient with agrammatism but without speech motor problems; [2] agrammatic patients with speech motor problems; and [3] aphasic patients without agrammatism but with speech motor problems. In our sample, we found 14 patients with both agrammatism and motor speech disorders, six patients with agrammatism only, and nine patients with motor speech disorder but without agrammatism.

This subdivision of the nonfluent/agrammatic PPA variant can be useful. In our experience, only patients with motor speech alterations developed, with disease progression, motor syndromes such as corticobasal degeneration or progressive supranuclear palsy (report in press).

The more recently recognized variant - PPA-L - has as core symptoms, described in the consensus, characteristics that are not exclusive. The first core feature - impaired single-word retrieval in spontaneous speech and naming - is a characteristic present in all the variants. Anomia is considered a universal symptom of the aphasias. The second core feature of PPA-L - impaired repetition of phrases and sentences - again cannot be considered exclusive to this variant. PPA-G cases can also present difficulties in repetition tasks. This absence of exclusive characteristics for the diagnosis of PPA-L can lead to a classification by exclusion. Our cases that were classified as PPA-L, besides fitting the characteristics in the consensus criteria, also presented the profile of PPA-L described by Gorno-Tempini et al. and by Henry & Gorno-Tempini.^26,27^ The diagnosis of PPA-L requires more attention and experience in the identification and recognition of the symptoms which are sometimes less evident. Furthermore, the two core and the ancillary features for classifying PPA-L also allow the inclusion of patients who do not present the specific profile defined by Gorno-Tempini et al. and Henry & Gorno-Tempini.^26,27^ For example, in our sample, two unclassifiable cases (PPA-U) fitted the consensus criteria for PPA-L. They had two core features (impaired single-word retrieval and impaired repetition) and three ancillary features (phonemic paraphasias, spared motor speech and absence of agrammatism). However, we preferred to consider them unclassifiable PPA (PPA-U), because their oral production constituted a form of neologistic jargon, similar to the oral expression seen in Wernicke's aphasics. The possibility of diagnostic criteria overlap leading to uncertainties regarding the precision of classification into one of the three variants, was discussed by Mesulam et al.^32^

As PPA-L does not have symptoms that are exclusively associated with this variant, we propose that the ancillary criteria "spared motor speech" and "absence of frank agrammatism" should be made core features in addition to those already included in the diagnostic criteria by Gorno-Tempini et al.^7^ This change would probably eliminate the possibility of a patient meeting criteria for both PPA-G and PPA-L. "Absence of neologist jargon" could also be included as an ancillary feature.

In the PPA clinical consensus report, Gorno-Tempini et al.^7^ argue that only a minority of cases present isolated symptoms or mixed characteristics, but with disease progression, the characteristics of one of the proposed variants became clearer, making classification possible. In the present study, after a cross-sectional evaluation, 20% of the cases were found not to fit any of the three variants. Hence, in our study and similarly in other reports,^15,31,32,46^ there are patients whose clinical characteristics do not fit the tripartite system proposed by the consensus. Mesulam et al.,^32^ in an analysis of 25 patients with early and mild PPA, were able to classify around 80% of the sample into one of the three variants.^32^ In the study of Sajjadi et al.^46^, in which 46 PPA patients were prospectively studied with the objective of classifying them into subtypes according to the consensus criteria, less than 60% of the sample was classifiable into any one of the PPA variants.^46^ By contrast, Leyton et al. classified 96% (45/47) of their PPA patients into one of the three subtypes.^15^

Unclassifiable cases could reflect a point during the evolution of the pathological process, and this concern was discussed by the consensus. However, we believe this explanation does not hold for all our unclassifiable cases, and the tripartite system is probably insufficient to classify the clinical and language characteristics of all PPA cases.

The follow-up of some of our cases also did not allow us to fit some patients into any of the proposed subtypes. Of the three cases of pure word deafness, two were accompanied longitudinally and presented with syntactic alterations in written production and surface dysgraphia with disease progression. However, we believe that despite the emergence of agrammatism, the most salient and dysfunctional symptom was difficulty in auditory/oral comprehension. Therefore, it would not have been congruent to label these cases as PPA-G, given the subsequent emergence of syntactic alterations with disease progression.

Mesulam et al. suggested the existence of a fourth variant of PPA, the mixed subtype (PPA-M), for cases that present syntactic and word comprehension impairment even in early and mild PPA.^31,32^ In our sample, only one unclassifiable case presented this PPA-M phenotype.

Mesulam et al. also found cases that presented only anomia and suggested the possibility that this clinical form could be a prodromic stage of PPA-S or be another PPA subtype, anomic variant (PPA-A).^32^ In our sample, six unclassifiable cases presented only anomia (PPA-A). In the follow-up, one of our cases developed characteristics of the logopenic and semantic variants concomitantly.

Follow-up studies can help define and better understand the different variants of PPA. Perhaps, the consensus classification needs some adjustments to accommodate cases that do not fit into any of the variants and to prevent overlap where cases fit more than one variant. Nonetheless, the established current guidelines are a useful tool to address the classification and diagnosis of PPA and are also of great value in standardizing terminologies to improve consistency across studies from different research centers.

## References

[1] Mesulam MM (1982). Slowly progressive aphasia without generalized
dementia. Ann Neurol.

[2] Harciarik M, Kertesz A (2011). Primary progressive aphasias and their contribution to the
contemporary knowledge about the brain-language relationship. Neuropsychol Rev.

[3] Mesulam MM, Grossman M, Hillis A, Kertesz A, Weintraub S (2003). The core and halo of primary progressive aphasia and semantic
dementia. Ann Neurol.

[4] Knibb JA, Hodges JH (2005). Semantic dementia and primary progressive aphasia: a problem of
categorization. Alzheimer Dis Assoc Disord.

[5] Adlam ALR, Patterson K, Rogers TT (2006). Semantic dementia and fluent primary progressive aphasia: two
sides of the same coin?. Brain.

[6] Rogaslki E, Mesulam M (2007). An update on primary progressive aphasia. Curr Neurol Neurosci Rep.

[7] Gorno-Tempini ML, Hillis AE, Weintraub S (2011). Classification of primary progressive aphasia and its
variants. Neurology.

[8] Mesulam MM (2001). Primary progressive aphasia. Ann Neurol.

[9] Mesulam MM (2003). Primary progressive aphasia - a language-based
dementia. New Engl J Med.

[10] Gorno-Tempini ML, Dronkers NF, Rankin KP (2004). Cognition and Anatomy of three variants of primary progressive
aphasia. Ann Neurol.

[11] Desgranges B, Matuszewski V, Piolino P (2007). Anatomical and functional alterations in semantic dementia: a
voxel-based MRI and PET study. Neurobiol Aging.

[12] Knibb JA, Xuereb JH, Patterson K, Hodges JR (2006). Clinical and pathological characterization of progressive
aphasia. Ann Neurol.

[13] Hodges JR, Mitchell J, Dawson K (2010). Semantic dementia: demography, familial factors and survival in a
consecutive series of 100 cases. Brain.

[14] Grossman M (2010). Primary progressive aphasia: clinicopathological
correlations. Nat Rev Neurol.

[15] Leyton CE, Villemagne VL, Savage S (2011). Subtypes of progressive aphasia: application of the International
Consensus Criteria and validation using β-amyloid
imaging. Brain.

[16] Nespoulous JL, Lecours AR, Lafond D, Parente MAMP (1986). Protocole Montréal-Tolouse MT-86 d'examen linguistique de
l'aphasie - version Beta.

[17] Goodglass H, Kaplan E (1993). The Assessment of aphasia and related disorders.

[18] Kaplan E, Goodglass H, Weintraub S (1983). The Boston Naming Test.

[19] Parente MAMP, Hosogi ML, Delgado AP, Lecours AR (1992). Protocolo de Leitura para o projeto H. F.S. P.(Human Frontier Science
Program).

[20] Senaha MLH, Caramelli P, Porto CS, Nitrini R (2007). Verbal and non-verbal semantic impairment: from fluent primary
progressive aphasia to semantic dementia. Dement Neuropsychol.

[21] Senaha MLH, Caramelli P, Porto CS, Nitrini R (2007). Semantic dementia Brazilian study of nineteen
cases. Dement Neuropsychol.

[22] Darley FL, Aronson AE, Brown JR (1975). Motor speech disorders.

[23] Agniel A, Joanette Y, Dojon B, Duchein C (1992). Protocole d'évaluation des gnosies visuelles.
Montréal-Toulouse.

[24] Howard D, Patterson K (1992). Pyramids and palm trees: a test of semantic access from pictures and
words.

[25] Otsuki M, Soma Y, Sato M, Homma A, Tsuji S (1998). Slowly Progressive Pure Word Deafness. Eur Neurol.

[26] Gorno-Tempini ML, Brambati SM, Ginex V (2008). The logopenic/phonological variant of primary progressive
aphasia. Neurology.

[27] Henry ML, Gorno-Tempini ML (2010). The logopenic variant of primary progressive
aphasia. Curr Opin Neurol.

[28] Rohrer JD, Rossor MN, Warren JD (2009). Neologistic jargon aphasia and agraphia in primary progressive
aphasia. J Neurol Sci.

[29] Folstein MF, Folstein SE, Mchugh PR (1975). Mini-mental state. A practical method for grading the cognitive
state of the patients for the clinician. J Psychiatry Res.

[30] Brucki SM, Nitrini R, Caramelli P, Bertolucci PH, Okamoto IH (2003). Suggestions for utilization of the mini-mental state examination
in Brazil. Arq Neuropsiquiatr.

[31] Mesulam M, Wieneke C, Rogalski E, Cobia D, Thompson C, Weintraub S (2009). Quantitative template for subtyping primary progressive
aphasia. Arch Neurol.

[32] Mesulam MM, Wieneke C, Thompson C, Rogalski E, Weintraub S (2012). Quantitative classification of primary progressive aphasia at
early and mild impairment stages. Brain.

[33] Mesulam MM, Weintraub S, Boller F (1992). Primary progressive aphasia: sharpening the focus on a clinical
syndrome. Heterogeneuty of Alzheimer's disease.

[34] Westburry C, Bub D (1997). Primary progressive aphasia: a review of 112
cases. Brain Lang.

[35] Clark DG, Charuvastra A, Miller BL, Shapira JS, Mendez MF (2005). Fluent versus nonfluent primary progressive aphasia: a comparison
of clinical and functional neuroimaging features. Brain Lang.

[36] Thompson CK, Cho S, Hsu CJ (2012). Dissociations Between Fluency And Agrammatism In Primary
Progressive Aphasia. Aphasiology.

[37] Rogalski ED, Johnson N, Weintraub S, Mesulam M (2008). Increased Frequency of Learning Disability in Patients With
Primary Progressive Aphasia and Their First-Degree Relatives. Arch Neurol.

[38] Graff-Radford J, Josephs KA (2012). Primary progressive aphasia and transient global
amnesia. Arch Neurol.

[39] Nitrini R, Hosogi-Senaha ML, Caramelli P (2012). Primary Progressive Aphasia and Transient Global
Amnesia. Arch Neurol.

[40] Ratnavalli E, Brayne C, Dawson K, Hodges JR (2002). The prevalence of frontotemporal dementia. Neurology.

[41] Rosso SM, Donker Kaat L, Baks T (2003). Frontotemporal dementia in The Netherlands: patient
characteristics and prevalence estimates from a population-based
study. Brain.

[42] Harvey RJ, Skelton-Robinson M, Rossor MN (2003). The prevalence and causes of dementia in people under the age of
65 years. J Neurol Neurosurg Psychiatry.

[43] Johnson JK, Diehl J, Mendez MF (2005). Frontotemporal lobar degeneration: demographic characteristics of
353 patients. Arch Neurol.

[44] Grossman M (2012). The non-fluent/agrammatic variant of primary progressive
aphasia. Lancet Neurol.

[45] Tan YE, Ng A, Kandiah N (2013). Frontotemporal Dementia in Southeast Asia: a comparative
study. Dement Geriatr Cogn Disord Extra.

[46] Sajjadi SA, Patterson K, Arnold RJ, Watson PC, Nestor PJ (2012). Primary progressive aphasia. A tale of two syndromes and the
rest. Neurology.

[47] Senaha MLH, Caramelli P, Nitrini R, Charchat-Fichman H, Weekes BS (2006). Semantic dementia without surface dyslexia. Brain Lang.

[48] Wilson MA, Martínez-Cuitiño M (2012). Semantic dementia without surface dyslexia in Spanish: unimpaired
reading with impaired semantics. Behav Neurol.

